# The Cross-Scale Association between Pathomics and Radiomics Features in Immunotherapy-Treated NSCLC Patients: A Preliminary Study

**DOI:** 10.3390/cancers16020348

**Published:** 2024-01-13

**Authors:** Abdou Khadir Dia, Leyla Ebrahimpour, Sevinj Yolchuyeva, Marion Tonneau, Fabien C. Lamaze, Michèle Orain, Francois Coulombe, Julie Malo, Wiam Belkaid, Bertrand Routy, Philippe Joubert, Philippe Després, Venkata S. K. Manem

**Affiliations:** 1 Department of Mathematics and Computer Science, Université du Québec à Trois Rivières, Trois-Rivières, QC G8Z 4M3, Canada; 2 Quebec Heart & Lung Institute Research Center, Québec City, QC G1V 4G5, Canadafabien.lamaze.1@ulaval.ca (F.C.L.); michele.orain@criucpq.ulaval.ca (M.O.); philippe.joubert@criucpq.ulaval.ca (P.J.); philippe.despres@phy.ulaval.ca (P.D.); 3 Department of Physics, Laval University, Quebec City, QC G1V 0A6, Canada; 4 Centre de Recherche du CHU de Québec-Université Laval, Quebec City, QC G1V 0A6, Canada; 5 Department of Molecular Biology, Medical Biochemistry and Pathology, Laval University, Quebec City, QC G1V 0A6, Canada; 6 Lille Faculty of Medicine, University of Lille, 59020 Lille, France; 7 Centre de Recherche du Centre Hospitalier Universitaire de Montréal, Montréal, QC H2X 0A9, Canada

**Keywords:** radiomics, pathomics, immunotherapy, non-small cell lung cancer

## Abstract

**Simple Summary:**

This study investigates the association between routine medical imaging and digitalized scans in lung cancer patients treated with immunotherapy. It involves analyzing features extracted from CT scans and histology images of 36 patients to understand how these different types of medical data correlate with patient survival and immune responses. The findings reveal significant connections between the imaging results and key health indicators, suggesting that combining these data types could improve personalized treatment strategies for lung cancer.

**Abstract:**

Background: Recent advances in cancer biomarker development have led to a surge of distinct data modalities, such as medical imaging and histopathology. To develop predictive immunotherapy biomarkers, these modalities are leveraged independently, despite their orthogonality. This study aims to explore the cross-scale association between radiological scans and digitalized pathology images for immunotherapy-treated non-small cell lung cancer (NSCLC) patients. Methods: This study involves 36 NSCLC patients who were treated with immunotherapy and for whom both radiology and pathology images were available. A total of 851 and 260 features were extracted from CT scans and cell density maps of histology images at different resolutions. We investigated the radiopathomics relationship and their association with clinical and biological endpoints. We used the Kolmogorov–Smirnov (KS) method to test the differences between the distributions of correlation coefficients with the two imaging modality features. Unsupervised clustering was done to identify which imaging modality captures poor and good survival patients. Results: Our results demonstrated a significant correlation between cell density pathomics and radiomics features. Furthermore, we also found a varying distribution of correlation values between imaging-derived features and clinical endpoints. The KS test revealed that the two imaging feature distributions were different for PFS and CD8 counts, while similar for OS. In addition, clustering analysis resulted in significant differences in the two clusters generated from the radiomics and pathomics features with respect to patient survival and CD8 counts. Conclusion: The results of this study suggest a cross-scale association between CT scans and pathology H&E slides among ICI-treated patients. These relationships can be further explored to develop multimodal immunotherapy biomarkers to advance personalized lung cancer care.

## 1. Introduction

Immune checkpoint inhibitors have significantly changed the therapeutic landscape of advanced non-small cell lung cancer (NSCLC) patients. Compared to conventional chemotherapy drugs, the integration of immunotherapy into treatment protocols has led to improved patient outcomes [1]. Despite their success, clinical benefit remains limited to only a subset of patients, and efforts are underway to build better immunotherapy biomarkers. In this regard, there has been an unprecedented increase in the availability of medical images and digitized pathological slides for cancer patients. Thus, these orthogonal data modalities provide a significant opportunity to aggregate and analyze them for the development of a new class of data-driven biomarkers. In the context of immunotherapy, their roles extend beyond diagnostics, monitoring of treatment responses, and early detection of immune-related adverse events [2,3]. Recent research efforts have been directed towards establishing radiomics and pathomics methodologies for developing more objective measurements and approaches to the clinical management of lung cancer [4,5,6].

Radiomics aims to focus on the development of novel biomarkers derived from radiological images [7,8,9]. The radiological features from the medical images contain information about biological processes that are extracted in a high-throughput manner. Radiomics can be quantified using well-defined mathematical equations to obtain the underlying characteristics of the disease through image intensity, shape, or texture, thereby overcoming the subjective nature of image interpretation [10]. Many studies, including our recent works, have investigated and reported on the added clinical value of radiomics features to predict various clinical and biological outcomes in immunotherapy-treated patients, such as overall survival, progression-free survival, tumor histology, and genetic profiling, among other endpoints [9,11,12,13]. On the other hand, the rapidly expanding field of pathomics aims to investigate the micro-scale patterns from the digitized histopathology slides, or whole slide imaging (WSI), in a high-throughput manner [4,14]. This is carried out to define many phenotypic aspects of cancer and patient risk in a variety of organs, including the lung [4,15,16]. Advances in scanning technology and the availability of large datasets of digital images have made it possible to explore the potential of computational image analysis for biomarker discovery [17,18,19]. The pathomics features encompass many characteristics of the tumor, such as morphology, intensity, texture, higher-order features, and spatial information. Several open-source libraries facilitate the development of computational pathology workflows. One such platform is Histolab, which is designed to handle WSIs, automatically detect the tissue, and enable testing alternative tiling strategies, including random extraction of tiles according to tissue detection score thresholds [20,21,22,23]. In recent years, many studies have utilized WSIs to develop novel biomarkers for patient survival and response [16,24,25].

The basic premise of integrating multimodal data types is that these modalities complement each other by augmenting the biological signals from individual data modalities to improve the inference of patients’ outcomes. The multimodal data integration from these two distinct imaging scales will definitely offer new research avenues to develop therapeutic biomarkers for immunotherapy-treated lung cancer patients. Furthermore, these integrative models will also help obtain insights into the biological features of tumors, such as intra-tumor heterogeneity. With the availability of medical imaging data (both radiological and WSI) in the context of immunotherapy, there are many challenges that are inherent to the multimodal integration of clinical cancer data, and some of them are more methodological [26]. For example, what is the inherent relationship between features generated from these multimodal datasets? Can the developed multimodal-derived models improve clinical outcomes for patients? Do they provide novel insights into the clinical and biological features that are complementary in nature? [4,5,27]. There are several works in the literature that show the ability of radiomics and pathomics to predict disease outcomes among NSCLC patients treated with immunotherapy; however, there is a paucity of studies on the combined role of radiomics and pathomics in inferring a patient’s response and survival. To achieve this, it is crucial to understand the underlying functional relationships between radiomics and pathomics features.

To date, few studies have explored the cross-scale association between radiomics and pathomics features in glioblastoma multiforme (GBM) and lung cancer. Recently, in a study led by Alvarez-Jimenez et al., the authors investigated the radiopathomics association between cellular density and image heterogeneity across radiology and histopathology datasets in lung cancer. This work demonstrated that the spatial patterns of tumor heterogeneity between these two approaches may be similar [4]. In another study [5], the authors determined the association between radiomic features extracted from preoperative ADC maps and post-contrast T1 images and pathomics features arising from H&E digitized pathology images in GBM patients. However, there has been a lack of studies investigating the relationship between radiomics and pathomics features in immunotherapy cohorts. With this premise, this work presents preliminary findings of the radiopathomics association in NSCLC patients treated with immunotherapy. Through this, we can identify pathomics features that may reflect the tissue composition basis of CT radiomics descriptors. Furthermore, we also explored the association of imaging-based features with clinical and biological endpoints—overall survival, progression-free survival, and CD8 immune cell counts.

## 2. Materials & Methods

### 2.1. Description of Cohorts

This retrospective study was performed on cohorts from two lung cancer centers: the Institut Universitaire de Cardiologie et de Pneumologie de Québec (Quebec Heart and Lung Institute, IUCPQ-Université Laval) and the Centre Hospitalier Universitaire de Montréal (CHUM). The corresponding institutional ethics committees have approved this project (MP-10-2020-3397). All patients who were diagnosed with advanced non-small-cell lung cancer and have received immune checkpoint inhibitor treatment have been included in this study. Accordingly, in these patients, we compiled survival endpoints and cell counts of the immunohistochemistry (IHC) marker, CD8 lymphocyte T phenotype. The progression of the disease was qualitatively evaluated by the radiologist. All the samples used in this study were part of the Quebec Respiratory Health Network Tissue Bank (https://rsr-qc.ca/biobanque/ accessed on 15 November 2020) at the IUCPQ. Informed consent was obtained from all the participants. In these cohorts, at least one pre-treatment CT scan (2 months before the administration of ICIs) and one post-treatment CT scan were available to assess disease progression following the treatment. Patients were treated with one of the three ICI compounds—Atezolizumab, Nivolumab, or Pembrolizumab. H&E slides were scanned on a NanoZoomer 2.0-HT slide scanner at 20X objective. A total of 24 and 12 samples from the CHUM and IUCPQ cohorts had both radiological data and H&E tissue slides. Overall, the collective dataset from these two centers includes a total of 36 patients for the downstream analyses.

### 2.2. Clinical and Biological Endpoints

Progression-free survival (PFS) represents the duration from the start of treatment until either the time when disease progression is observed, the date of death due to any reason, or the last follow-up date (censored). The overall survival (OS) rate is computed from the date of the patient’s diagnosis to either the date of death from any cause or the date of censoring, whichever occurs first [28]. Imaging techniques like CT scans, PET-CT scans, and MRIs are commonly employed to confirm disease advancement [11,12].

CD8 (cluster of differentiation 8) is a transmembrane glycoprotein that functions as a co-receptor for the T-cell receptor (TCR). The CD8 co-receptor serves a role in T cell signaling and facilitates interactions between cytotoxic T cells and antigens. CD8 T cells were evaluated to determine the status of immune infiltration in tumor samples. The CaloPix image analysis software (Tribun Health) was used to score IHC slides, with the results expressed as % of positive cells’ surface per square micrometer of tumor (μm^2^).

### 2.3. Radiomics Features Analysis

To extract features from the radiological scans, it is crucial to normalize the slice thicknesses of all CT images, which was done by interpolating them to a voxel size of 1 × 1 × 1 $mm^{3}$ through the B-spline interpolation method available in the SimpleITK (Simple Insightful Toolkit) library. Normalizing the slice thicknesses was essential to ensure consistency and standardized representation, achieve voxel alignment, and improve the accuracy of feature calculations. These measures contribute to reliable and comparable results, enabling meaningful comparisons across different patients and imaging studies. We used the Pyradiomics version 3.0.1 computational platform in Python [29] to extract the features from the pre-treatment CT scans. Pyradiomics is an open-source software library that allows for the extraction of radiomics features from medical imaging data in any format readable by ITK compliant with the image biomarker standardization initiative (IBSI https://arxiv.org/abs/1612.07003 accessed on 20 October 2020) [30]. This platform also provides several built-in features for pre-processing, such as normalization and resampling.

Radiomics features are generally categorized into three main groups based on their properties and calculation methods: (i) morphological or shape-based features—these features pertain to the shape and structure of the image, calculated in both 2D and 3D views. This category includes a subset of attributes describing the image’s shape, like volume, surface area, compactness, and sphericity; (ii) first-order or intensity-based features—these features quantify the intensity characteristics of the tumor based on the statistical properties of tumor intensity distribution within the region of interest (ROI). They include metrics like mean, median, and mode, along with indicators of distribution symmetry and diversity such as percentiles, skewness, kurtosis, and entropy; and (iii) second-order or texture-based features—these features consider the statistical dependencies and spatial relationships between adjacent voxels. Several methods are used to figure them out, including the gray-level co-occurrence matrix (GLCM), the gray-level run-length matrix (GLRLM), the gray-level size zone matrix (GLSZM), the gray-level distance zone matrix (GLDZM), and the neighborhood gray-tone difference matrix (NGTDM). For instance, GLCM quantifies the occurrence of pairs of pixel intensities at different spatial distances and orientations within the ROI, presenting them in matrix form. In addition, filter-based (e.g., wavelet, Laplacian of Gaussian, logarithm, exponential) radiomics features can be extracted from derived images using filters that can be applied to both texture- and intensity-based features.

### 2.4. Whole Slide Image (WSI) Pre-Processing

Pre-processing of WSIs is a crucial preliminary procedure before patch extraction, and various anomalies may occur during the process of digitizing WSIs. These anomalies observed in WSIs encompass various artifacts, such as noise and background interference. Prior to patch extraction, a thorough pre-processing of the WSIs is essential to methodically identify regions of interest. The ‘histolab’ module in Python was employed to carry out the WSI pre-processing methods. In order to clearly define the tissue region while eliminating artifacts and background interference, a series of procedures were implemented. These procedures consist of the following steps: conversion of the image to grayscale, application of Otsu thresholding, binary dilation, elimination of small holes, removal of small objects, etc. By using the aforementioned procedure, we obtained WSIs (without noise and artifacts) that exhibit a minimum of 80% tissue content. This procedure ensured that only the relevant part of the WSIs, known as the region of interest, was preserved for the downstream analyses. As the goal of this study was to investigate the relationship between radiomics features, pathomics features, and clinical endpoints, it was critical to consider only those areas of the WSI that have a significant number of nuclei.

This focus on regions with a substantial number of nuclei is driven by the need to ensure that the extracted patches are representative of the tissue’s cellular structure and pathology. Therefore, the pre-processing steps were designed to enhance the visibility and contrast of the nuclei within the tissue, facilitating their accurate identification and analysis. Moreover, this meticulous pre-processing approach aids in minimizing the potential biases that could arise from non-tissue elements or artifacts present in the WSIs. By concentrating on areas rich in nuclei, the study aims to establish a more robust and direct correlation between the extracted features and the clinical endpoints, thereby providing more reliable and clinically relevant insights. This careful selection of regions for analysis is not only crucial for the accuracy of the study but also enhances the reliability of the conclusions drawn regarding the interplay of radiomics and pathomics features in the context of clinical outcomes.

### 2.5. WSI Segmentation and Patching

The extracted patches from the WSI measure 512 × 512 pixels with a 20× magnification. All patches of this size containing a nucleus percentage of 15% or higher were extracted [31]. The extraction of these patches was accomplished using the ScoreTiler class from histolab (available online: https://histolab.readthedocs.io/en/latest/readme.html, accessed on 20 October 2020). The ScoreTiler class computes a score for each patch extracted from the WSI based on the detected nuclei count. It incorporates multiple techniques like threshold-based techniques, color space conversion [32,33], watershed transformation [34,35], and morphological operations [36,37] to facilitate nucleus detection. Patches were subsequently divided into bins of different scales (16 × 16 px, 32 × 32 px, and 64 × 64 px). The estimation of cell density, which measures the spatial arrangement of cells within a tissue, is conducted for individual patches at a specific scale, as previously described by Alvarez-Jimenez et al. [4]. The cell density was determined by the segmentation of nuclei and the allocation of a certain gray level to each patch, which was determined by the estimated number of nuclei contained within that region [4,5]. The resulting output consists of a map that displays the density of cells at different scales, providing a visual representation of the distribution of cell density across different areas within the patch. The approach utilized for nucleus segmentation in this study was the Watershed algorithm [35,38]. Prior to using the algorithm, several preprocessing stages were conducted on the image patches. We removed small white noises present in the image, as well as small holes in the object (nuclei), to ensure that the region near the center of the object was considered as the foreground region, while those farther away from the object were regarded as the background. Subsequently, the area that represents the nuclei was identified by using the distance transform with an appropriate threshold. We then isolated the area that was not the nuclei by applying a dilation function, which shifts the object’s boundary towards the background [39]. Following the isolation process, the Watershed algorithm was employed. This algorithm functions by discerning the high and low points in the grayscale image, thus effectively outlining each nucleus, even within regions where they are densely packed. Such segmentation is pivotal for accurately estimating cell density, as it facilitates the precise identification and enumeration of individual nuclei in each patch. Post-segmentation, the nuclei were categorized according to their size and shape attributes, enhancing the analysis of cell density. This categorization is crucial for differentiating among various cell types and conditions, which is essential for a deeper comprehension of the biological activities in the tissue. Employing this combination of techniques allows for an exhaustive and nuanced examination of the tissue microenvironment, yielding critical insights beneficial for both diagnostic and research objectives.

### 2.6. Extraction of Pathomics Features

Haralick features constitute a collection of statistical descriptors employed to characterize the texture or patterns discernible within an image. These features were derived from the gray-level co-occurrence matrix (GLCM), which is a matrix recording the frequency of occurrences of distinct pairs of pixel intensity values at predefined relative positions within an image. The utilization of Haralick features in the analysis of cell density maps enables a quantitative depiction of the spatial distribution and patterns of cell density. These features were obtained from each patch for each scale. From each matrix of cell density values, four co-occurrence matrices were computed for each scale (horizontal, vertical, minor diagonal, and main diagonal).

### 2.7. Radiopathomics Analysis

**Figure 1:**
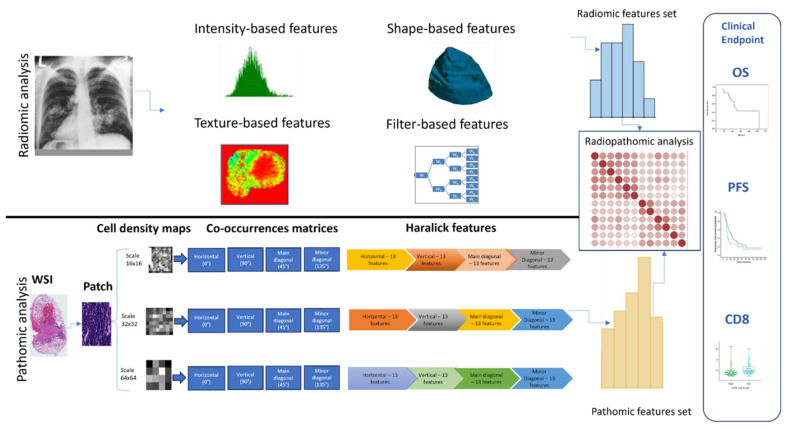
Process flow of the radiopathomics evaluation employed in this research: The upper row illustrates the sequential radiomics analysis process, from initial radiological imaging to feature extraction and statistical analysis. The lower row details the corresponding pathomics analysis, starting from histological slide preparation to intricate feature extraction, with both pathways converging on the assessment of clinical endpoints like OS (Overall Survival), PFS (Progression-Free Survival), and CD8 cell count distributions.

To investigate a possible association between radiomics and pathomics features in immunotherapy-treated NSCLC patients, an integrative study design was developed as presented in Figure 1. The initial row delineates the steps carried out on the pre-treatment CT scans for radiomics analysis, while the subsequent row outlines the steps used for pathomics analysis. After performing radiomics and pathomics feature extraction, a comprehensive analysis was conducted to explore the relationship between radiomics and pathomics, radiomics and clinical outcomes (overall survival, progression-free survival, and CD8), and pathomics and clinical outcomes (overall survival, progression-free survival, and CD8). The association between imaging-derived features was computed using the Spearman correlation. In order to evaluate the variations in correlation distributions between radiomics and pathomics features and clinical outcomes, the Kolmogorov-Smirnov (KS) test was utilized. This statistical measure, obtained from the scipy.stats package in Python, is employed to quantify the similarity between two distributions.

### 2.8. Feature Reduction Analysis

Initially, data normalization was done by subtracting the mean and dividing by the standard deviation. This type of data normalization, referred to as z-score normalization, is vital as it ensures that all data sets operate on the same scale, which is crucial for correlation studies. A correlation filter was then constructed to reduce feature redundancy. This correlation filter is based on Spearman’s correlation coefficient, which is known for its ability to detect monotonic relationships and its robustness against outliers [4]. A threshold of |r| = 0.9 was established, and all pairs of correlations between pathomics and radiomics features with an absolute correlation value greater than 0.9 were examined individually [5,40]. In summary, when two features exhibit the absolute value of the correlation coefficient (r) exceeding 0.9, the algorithm assesses the mean absolute correlation of each variable, and the variable with the highest mean absolute correlation is subsequently eliminated. Spearman’s correlation was also used to compute the correlation between radiomics features and clinical endpoints (OS, PFS, and CD8), as well as between pathomics features and clinical endpoints (OS, PFS, and CD8). To control for the false positives, we performed multiple hypothesis testing using the False Discovery Rate (FDR) method.

### 2.9. Hierarchical Clustering of Features

Unsupervised clustering using the hierarchical agglomerative clustering (HAC) methodology was employed to better understand the similarities and differences in terms of patient stratification for immune checkpoint inhibitors. HAC is a clustering technique that structures data based on their similarities by progressively building a clustering tree or dendrogram. This method was applied to the pathomics and radiomics features to group patients into clusters based on their respective data profiles. The algorithm was configured to divide the patients into two distinct clusters, resulting in the formation of two separate groups of patients. The division into two clusters was a crucial step in analyzing the clinical data, as it will facilitate the identification of significant distinctions between the groups.

## 3. Results

### 3.1. Patient Characteristics

Table 1 presents the study population demographics and their clinical characteristics. Continuous data was presented as mean ± standard deviation (SD), while categorical data was presented as counts and percentages. The cohort consists of 36 patients who have both radiological and WSI slides. The mean age of NSCLC patients was 67 years (7.2). 72% of the patients in the smoking category were former smokers, while 25% of them were current smokers. The range of OS values was 1.5–62 months, and the range of PFS values was 0.46–62 months. The average percentage of CD8 cell counts was estimated at 7.6%, with a standard deviation of 6.9%.

### 3.2. Feature Extraction and Reduction

Using the Pyradiomics pipeline, a total of 851 radiomics features were extracted from the annotated ROI of the pre-treatment CT scans. For the pathomics part, a total of 13 haralick features were computed for each co-occurrence matrix, resulting in a cumulative count of 156 features per patch. In order to obtain the features at the WSI level, five statistical measures (mean, median, variance, kurtosis, and skewness) were computed over all patches inside each WSI. Consequently, WSI is characterized by a total of 260 distinct attributes for each scale.

The removal of highly correlated variables will enhance the precision, stability, and interpretability of the correlation analysis between radiomics and pathomics features, which was crucial for obtaining reliable and informative results. Following the application of the correlation filter to remove redundant features, the number of pathomics features decreased from 260 to a total of 88, distributed as 17 features at the 16 × 16 px scale, 30 features at the 32 × 32 px scale, and 41 features at the 64 × 64 px scale. For the radiomics features, after applying the correlation filter, only 156 out of 851 extracted features were retained, with the other 695 being excluded due to their strong mutual correlation (|r| > 0.9). These sets of 88 and 156 pathomics and radiomics features were utilized for downstream analyses.

### 3.3. Association between Pathomics and Radiomics Features

**Figure 2:**
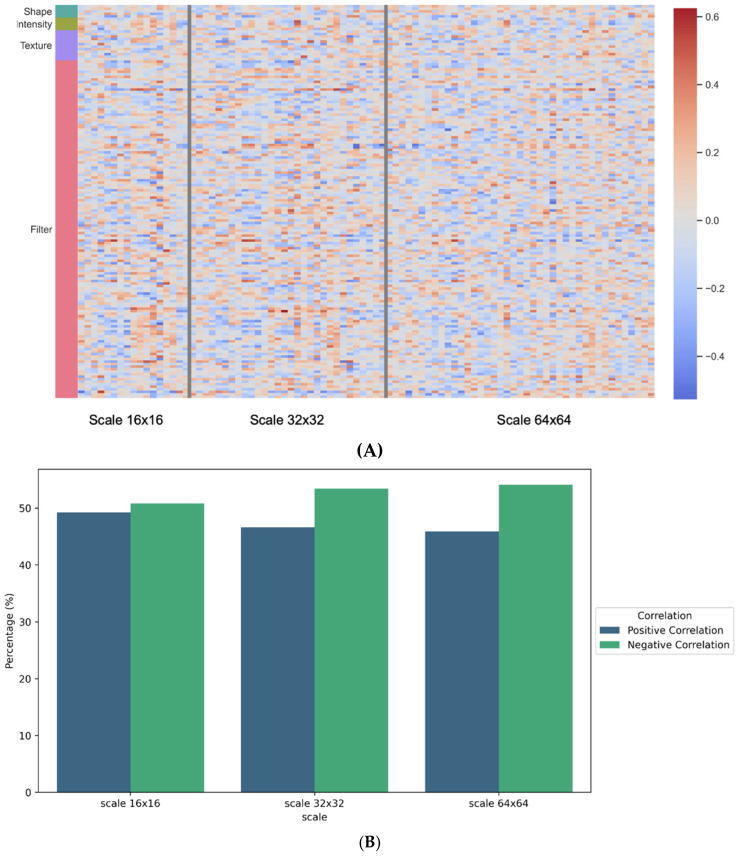
(**A**) Detailed correlation matrix heatmap. (**A**) Displaying Spearman’s Rank Correlation Coefficients between pathomics and radiomics features across multiple scales: The color gradient from red to blue represents the spectrum of positive to negative correlations, respectively, segmented by feature category (Shape, Intensity, Texture, and Filter) and spatial resolution (Scale 16 × 16, Scale 32 × 32, Scale 64 × 64). The intensity of the color indicates the strength of the correlation, providing insights into the multidimensional relationship between imaging characteristics and histopathological patterns. (**B**) Bar Chart illustrating the proportion of positive and negative correlations between radiomics and pathomics features at varied analytical scales: This chart quantitatively contrasts the prevalence of positive (blue) and negative (green) correlation coefficients, providing a clear visual representation of the directional relationships across distinct spatial resolutions in the dataset.

Figure 2A displays the correlation heatmap between pathomics and radiomics features, while Figure 2B illustrates the comparison of the percentage positive correlations and negative correlations. For the scale of 16 × 16 px, the range of correlation coefficient values was found to be between −0.60 and 0.58. Positive correlations account for 49% (i.e., 1314) and negative ones for 51% (i.e., 1355) of the total correlations. The most strongly correlated radiomics features were the wavelet features. On the pathomics side, features such as “sum variance, sum average, and Information Measure of Correlation 2” were predominant. For the scale of 32 × 32 px, correlation values ranged from −0.57 to 0.57. Positive correlations account for 47% (i.e., 2195) of the total, whereas negative correlations account for 53% (i.e., 2514). The wavelet features from the radiomics and the Information Measure of Correlation 1 and 2, sum average, and difference variance from the pathomics are the features that were most correlated to each other. For the 64 × 64 px scale, correlations between the features extracted from the cellular density map and the radiomics features range between −0.64 and 0.58. Negative correlations account for 54% of the total (3481), while positive correlations account for 2950. Wavelet features among the radiomics features were the most strongly associated with pathomics. To summarize our findings, we found significant cross-scale associations between radiomics- and pathomics-derived handcrafted features.

The significant radiopathomics association given by the highest correlation coefficient (r > 0.5) is presented in Table 2.

**Figure 3:**
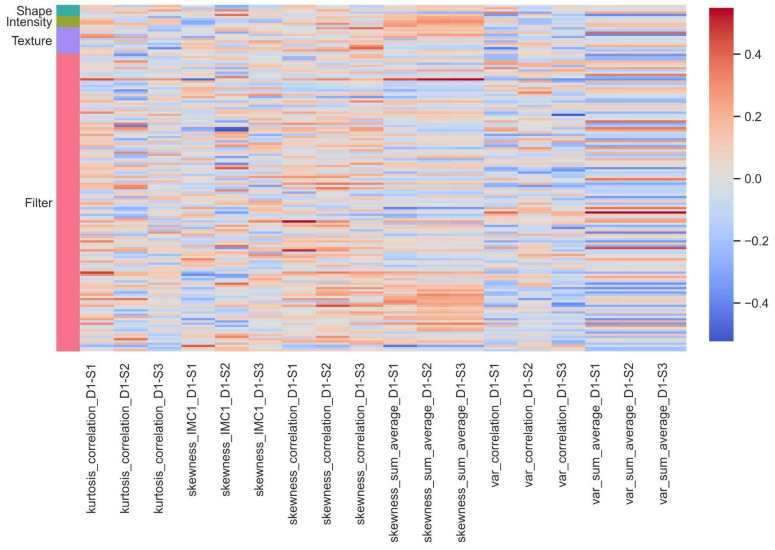
Detailed heatmap visualization of correlation patterns between radiomics categories and common pathomics features across all analytical scales: this comprehensive figure delineates the extent of correlation, with shades of red and blue representing the degree of positive and negative correlation, respectively. Each row corresponds to a specific feature, categorized by shape, intensity, texture, or filter, and each column represents the correlation of that feature with common pathomics features at different scales, offering a nuanced view of the interplay between radiological imaging attributes and pathological characteristics.

We also investigated the association of pathomics features with different groups of radiomics-based features. Figure 3 illustrates the heatmap of correlations between various groups of radiomics features and the common correlated pathomics features across all scales. In the variable naming, “S1” refers to the 16 × 16 scale, “S2” to the 32 × 32 scale, and “S3” also refers to the 64 × 64 scale. Filter-based features, as well as pathomics features like correlation, information measure of correlation 1, and sum average, were found to be significant in this association study. These findings suggest that there are meaningful relationships between textural and structural characteristics extracted from radiological and pathological data. The presence of correlations between these different types of features highlights the potential for synergistic information when combining radiomics and pathomics analyses, which can lead to a more comprehensive understanding of the underlying biological and clinical aspects of the studied NSCLC cases.

### 3.4. Association between Imaging Features and Survival Endpoints

**Figure 4:**
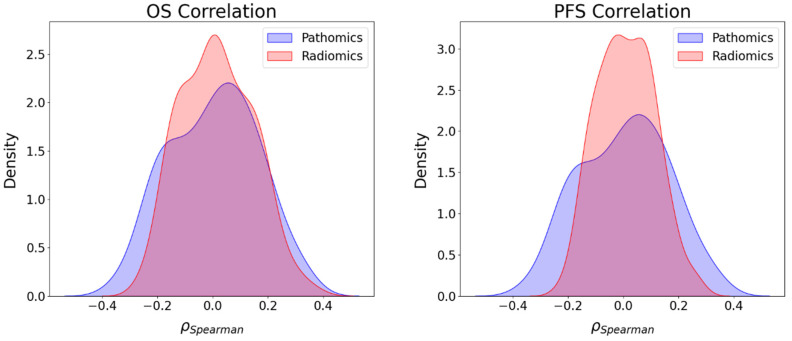
Correlation distribution between pathomics and radiomics features and survival endpoints (**left**—OS and **right**—PFS).

The association between imaging features, namely, radiological and pathomics with the two survival endpoints, PFS and OS, was examined. The range of the absolute value of the correlation coefficient between pathomics features and OS was found to be between −0.34 and 0.34, while for PFS, the range was between −0.32 and 0.33. Conversely, the correlation coefficients between radiomics features and OS ranged from −0.26 to 0.37, and for PFS, they ranged from −0.23 to 0.27 (Figure 4). Within the pathomics feature space, we found 54 and 34 features to be positively and negatively correlated with PFS, respectively, while 47 and 41 features were found to be positively and negatively correlated with OS, respectively. Similarly, within the radiomics latent space, we obtained 82 and 74 to be positively and negatively correlated with PFS, respectively, and 83 and 73 features to be positively and negatively correlated with the OS, respectively.

Furthermore, the KS test was employed to examine the similarity between the distribution of correlation values produced by radiomics and pathomics features and the survival endpoints. The KS test yielded a statistic of 0.202 with a *p*-value of 0.017 for comparing the distribution of radiomics and pathomics with PFS. The analysis revealed a significant difference in the distribution of correlation coefficients between radiomics and pathomics features concerning PFS. In contrast to PFS, the analysis of correlations with OS showed no strong evidence of a significant difference in the distributions of correlation coefficients. The KS statistic for OS was 0.137, with a *p*-value of 0.217. These results indicated that the distribution of the correlations between radiomics and pathomics features and OS is similar.

### 3.5. Association between Imaging Features and CD8 Counts

**Figure 5:**
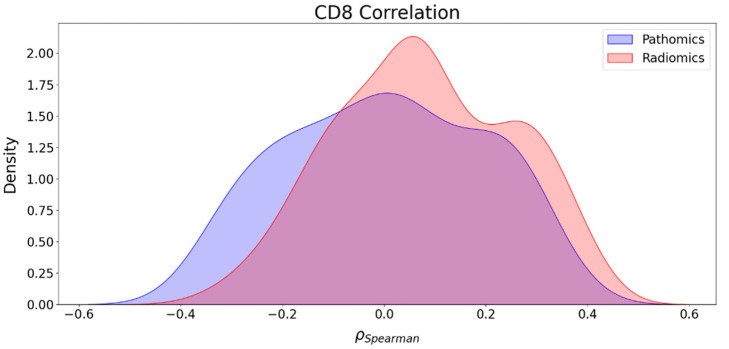
Correlation distribution between pathomics and radiomics features and CD8.

A study on the association between imaging features and CD8 endpoints was conducted. The analysis revealed that the correlation between pathomics features and CD8 ranges from −0.35 to 0.33, and in the case of radiomics features, it spans from −0.34 to 0.41, as illustrated in Figure 5. The number of positive and negative correlations between pathomics features and CD8 was the same, with 44 for each. In the case of radiomics features, 106 positive correlations were observed against 50 negative ones. To compare the two distributions, we performed the KS test. We obtained the KS statistic of 0.196 with a *p*-value of 0.022, suggesting that there is a statistically significant difference between the two distributions and that the two sets of imaging features exhibit a distinct association with the CD8 immune phenotype. The main variables influencing the correlations between pathomics features and clinical endpoints were identified as contrast, correlation, sum variance, sum average, and Information Measure of Correlation 1. However, the radiomics features most influencing correlations with clinical endpoints predominantly belonged to the filter-based features.

### 3.6. Clustering of Features

**Figure 6:**
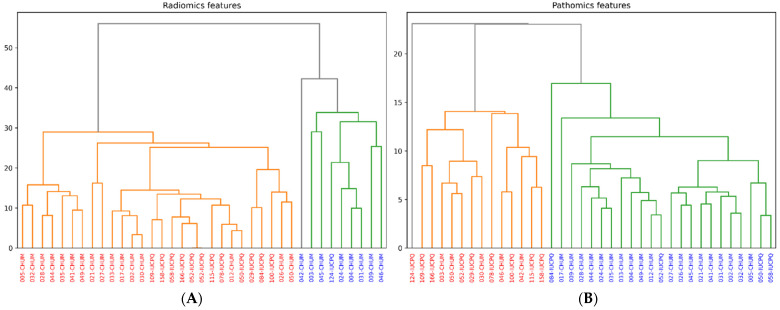
Hierarchical clustering analysis using. (**A**) pathomics features; (**B**) radiomics features.

We cut the dendrogram generated by the features of the two imaging modalities at a height such that we obtained two distinct clusters. The examination of the dendrograms below (Figure 6) reveals two distinct clusters, one based on pathomics features and the other on radiomics features. In the dendrogram of radiomic features, cluster 0 comprises 27 patients identified by red labels, while cluster 1 consists of 9 patients with blue labels. As for pathomics features, cluster 0 includes 12 patients, while cluster 1 contains 24. An interesting observation is that 22 patients changed clusters when transitioning from pathomics to radiomic features. For the pathomics features, patients grouped in cluster 0 exhibited higher PFS and OS compared to those patients in cluster 1. This observation suggests that cluster 0 may represent a group of patients with a better treatment response or slower disease progression than cluster 1. Similarly, for the radiomics features, the same scenario is observed in cluster 1, where patients show both higher PFS and OS compared to those in cluster 0. Furthermore, when examining the radiomics features, differences are noted in the proportion of patients with CD8 levels above the median between the two clusters. Cluster 1 has a higher proportion of patients with CD8 values above the median compared to Cluster 0. This suggests that the patients in cluster 1 may be more responsive to immunotherapy than the patients in cluster 0.

Furthermore, a more in-depth analysis of the significant differences between the clusters generated by radiomics and pathomics features is presented in Table 3.

## 4. Discussion

In the clinical management of cancer, a compendium of tests across different modalities is carried out by the patients. Through these tests, a variety of data sets are generated along the diagnostic and therapeutic trajectory of patients. A promising opportunity thus emerges for us to leverage these distinct datasets, analyze them, and build data-driven multimodal biomarkers to improve cancer care. For example, OMICS-based assay panels are being used in clinical care. And imaging modalities such as CT and pathological slides are being used significantly to build AI models for improving patient management in terms of diagnosis and interventions. This compendium of complementary datasets across larger patient cohorts opens up new research avenues to enhance clinical decision-making.

The fundamental idea of integrating disparate multimodal datasets is that they augment the biological information of a tumor and complement each other. This would improve the performance of the developed predictive models. In this context, radiological images and WSI’s describe the tumor characteristics at two different scales—micro and macro—thus inferring the underlying biological mechanisms of the phenotype. Therefore, a new class of multimodal biomarkers is required, harnessing information from various sources with refined predictive models that can improve the therapeutic response. There have been efforts to lead the development of radiomics and pathomics biomarkers to predict immunotherapy response and survival independently. In lung cancer, there are a few studies that focus on exploring the correlation between pathomics and radiomics features. In the study by Charlems et al. [4], the authors examined the correlation between Haralick-type features and radiological features, which were later used to classify different cancer subtypes. Wan et al. successfully predicted a favorable pathological response after neoadjuvant radiotherapy for locally advanced rectal cancer patients using a model that integrates radiomics and pathomics features [41]. Furthermore, Lu et al. provided a concise synthesis of the integration of pathomics, radiomics, and genomics, as well as an overview of research studies that have combined radiomics and pathomics, especially in the context of cancer prognosis [6,42]. However, there is a paucity of studies to build multimodal biomarkers integrating both radiomics and pathomics workflows in the context of immunotherapy. To achieve this, it is essential to understand the cross-sale association between radiomics and pathomics features among immunotherapy-treated NSCLC patients.

With this premise, we explored the association between radiomics and pathomics features (at different scales). The first part of our study focused on the analysis of the correlation between radiomics and pathomics features. Pathomics features were extracted from cell density maps at three levels of resolution. We focused exclusively on Haralick-type features for the analysis of histological images, following the approach employed by Alvarez et al. in their work [4]. Radiomics features were extracted from the pre-treatment CT scans, which are categorized into morphological, intensity-based, and texture-based features. Nevertheless, it is important to note that not all retrieved features were incorporated into the radiopathomics study. A correlation-based filtering method was employed to remove any redundant radiomics and pathomics features. In order to prevent multicollinearity and reduce the loss of information, we employed a threshold of 0.9, as suggested by Peeters et al. in their research on predictive analysis using radiomics data [40]. This filter allowed us to retain the most relevant variables for analysis. The correlation values obtained across the three different resolutions ranged from −0.64 to 0.58. Filter-based features, particularly wavelet features, proved to be the most influential in this analysis. On the pathomics side, the variables most correlated with wavelet features included sum variance, sum average, Information Measure of Correlation 1 and 2, and correlation.

Furthermore, we also investigated the association between radiomics and clinical endpoints (OS, PFS, and CD8) and between pathomics features and clinical endpoints (OS, PFS, and CD8). The results showed that the strongest correlations in absolute value between pathomics features and PFS, OS, and CD8 were 0.34, 0.33, and 0.35, respectively. For PFS and OS, positively associated correlation values were higher than negative correlations, while for CD8, the number of positive and negative correlations was balanced. Regarding radiomics features, the highest correlations in absolute value for PFS, OS, and CD8 were 0.27, 0.37, and 0.41, respectively. In addition, the KS test was utilized to assess the differences in correlation distributions between radiomics and pathomics features and clinical endpoints. In the case of the distribution of correlations with PFS and CD8, the KS statistics of 0.202 (*p*-value = 0.02) and 0.196 (*p*-value = 0.02) indicate distinct associations with these clinical endpoints, respectively. However, the analysis of the distribution of the correlations with OS showed no strong evidence of a significant difference in the distributions with KS statistics of 0.137 (*p*-value = 0.217). Furthermore, while both radiomics and pathomics features exhibit peaks at zero, the correlation distribution of pathomics features with PFS displays a lower maximum frequency at zero and extended tails. This suggests the existence of significant exceptions, where stronger correlations may be present compared to the radiomics features. These exceptions should be further explored with a larger sample size.

We have observed a more pronounced correlation in absolute value between wavelet features and certain Haralick-type features, such as “sum variance, sum average, correlation, and Information Measure of Correlation 1 and 2”. This can be attributed to the nature of these features, which inherently capture textural aspects of images. Wavelet features are particularly sensitive to abrupt changes in an image, such as edges or texture transitions. On the other hand, Haralick features, like “correlation and cumulative mean”, also describe texture and intensity relationships between adjacent pixels. Cell density maps can also identify histological or cytological changes at the microscopic level that are visible in radiological images. The correlations between radiomics, pathomics features, and clinical endpoints result from a complex interplay of several factors. Tumors are inherently heterogeneous, meaning different regions within a tumor exhibit distinct radiomics and pathomics characteristics and may impact the underlying relationship between radiomics and pathomics features. The hierarchical clustering allowed us to gain insight into patient stratification and its implications for immunotherapy treatment. By dividing patients into two clusters based on their pathomics and radiomics profiles, we aimed to uncover potential patterns in treatment response and disease progression. Irrespective of the imaging features, patients with better survival (PFS and OS) and CD8 counts were grouped into one cluster.

Despite promising findings in this work, we do acknowledge the limitations, which are: (i) potential cohort-specific biases in design; (ii) small sample size (*n* = 36); (iii) heterogeneity of CT scanners and staining variations across centers, along with the impact of gray level discretization on the radiomics feature extraction. These limitations can be partially addressed by the incorporation of harmonization approaches to combat variations induced by the scanner and staining protocols. While our work reveals interesting cross-scale relationships between radiology and pathology in immunotherapy-treated NSCLC patients, the findings warrant careful interpretation due to the small sample size. Altogether, discovering correlations between these multimodal features can enhance the use of medical imaging as an “in silico biopsy” to better manage immunotherapeutic interventions. This could potentially pave the way for personalized medicine using exclusively imaging data, which is more accessible than invasive biopsies.

## 5. Conclusions

This study highlights the potential of integrating radiomics and pathomics data in the treatment of non-small-cell lung cancer, especially in patients undergoing immunotherapy. By analyzing correlations between these multimodal imaging features and clinical outcomes, our research offers insights into the development of personalized treatment strategies. Despite limitations like the small sample size and variations in imaging techniques, our findings underscore the value of using combined imaging modalities to enhance patient care in oncology. This approach holds promise for advancing personalized medicine and optimizing immunotherapeutic interventions.

## Figures and Tables

**Table 1:** Clinical characteristics of non-small cell lung cancer patients treated with ICIs.

Clinical and Pathologic Characteristics	Value
Age [mean ± SD]	67 ± 7.2
Sex [*n* (%)] Male Female	13 (36%) 23 (64%)
BMI [mean ± SD]	25.7 ± 5.3
Smoking habit [*n* (%)] Current Former Never	9 (25%) 26 (72%) 2 (3%)
Progression [*n* (%)] Yes No	28 (76%) 8 (24%)
OS [*n* (%)] ≤12 months >12 months	8 (23%) 28 (77%)
PFS [*n* (%)] ≤12 months >12 months	26 (61%) 14 (39%)
CD8 [mean ± SD]	7.6 ± 6.9

**Table 2:** Summary of the highest-correlated (r > 0.5) radiomics-pathomics features.

Radiomic Feature Name	Pathomics Feature Name	Correlation	Scale
wavelet.HLL_glszm_GrayLevelVariance	kurtosis_IMC1_D3	−0.644	64 × 64
wavelet.HLH_gldm_DependenceEntropy	var_sum_average_D1	0.578	64 × 64
wavelet.LHL_firstorder_Mean	skewness_IMC2_D2	−0.568	64 × 64
wavelet.LLH_glszm_SmallAreaEmphasis	skewness_correlation_D3	−0.526	64 × 64
original_glszm_SmallAreaEmphasis	skewness_correlation_D3	−0.502	64 × 64
wavelet.HLL_gldm_DependenceEntropy	kurtosis_IMC1_D3	−0.500	64 × 64
wavelet.HLH_gldm_DependenceEntropy	var_sum_average_D1	0.578	32 × 32
wavelet.HLH_gldm_DependenceEntropy	kurtosis_IMC2_D3	−0.572	32 × 32
wavelet.LHH_firstorder_Mean	skewness_IMC1_D3	−0.537	32 × 32
wavelet.LHH_firstorder_Mean	skewness_contrast_D4	−0.527	32 × 32
original_ngtdm_Busyness	median_sum_variance_D2	−0.604	16 × 16
wavelet.HLH_gldm_DependenceEntropy	kurtosis_IMC2_D3	−0.551	16 × 16
wavelet.LHH_firstorder_Mean	var_IMC2_D1	0.521	16 × 16

**Table 3:** Unsupervised clustering of NSCLC patients using radiomics and pathomics features, independently.

Clusters Based on Pathomics Features				
Cluster	Cluster Size	Number (%) of Patients with PFS > 12 Months	Number (%) of Patients with OS > 12 Months	Number (%) of Patients with CD8 > Median
0	24	11 (46%)	18 (75%)	12 (50%)
1	12	3 (25%)	10 (83%)	6 (50%)
**Clusters based on radiomics features**				
0	9	3 (33%)	8 (89%)	3 (33%)
1	27	11 (41%)	20 (74%)	15 (56%)

## Data Availability

We would like to thank the IUCPQ research center for providing the data. Data presented in this study are not publicly available at this time but may be obtained from the corresponding author, Venkata Manem, upon reasonable request.
